# Under-sampling in epilepsy: Limitations of conventional EEG

**DOI:** 10.1016/j.cnp.2020.12.002

**Published:** 2020-12-30

**Authors:** Maxime O. Baud, Kaspar Schindler, Vikram R. Rao

**Affiliations:** a Sleep Wake Epilepsy Center, NeuroTec and Center for Experimental Neurology, Department of Neurology, Inselspital Bern, University Hospital, University of Bern, Switzerland; b Wyss Center for Bio- and Neuro-engineering, Geneva, Switzerland; c Department of Neurology and Weill Institute for Neurosciences, University of California, San Francisco, United States

**Keywords:** Interictal epileptiform activity, Chronic EEG, Chronobiology, Circadian, Multidien

## Abstract

The cyclical structure of epilepsy was recently (re)-discovered through years-long intracranial electroencephalography (EEG) obtained with implanted devices. In this review, we discuss how new revelations from chronic EEG relate to the practice and interpretation of conventional EEG. We argue for an electrographic definition of seizures and highlight the caveats of counting epileptiform discharges in EEG recordings of short duration. Limitations of conventional EEG have practical implications with regard to titrating anti-seizure medications and allowing patients to drive, and we propose that chronic monitoring of brain activity could greatly improve epilepsy care. An impending paradigm shift in epilepsy will involve using next-generation devices for chronic EEG to leverage known biomarkers of disease state.

## Highlights

• Interictal epileptiform activity displays wide fluctuations over time limiting the interpretability of conventional EEG.
• When accounting for these fluctuations, interictal epileptiform activity is a biormarker to prognosticate seizure recurrence.
• Treatment titration may be achievable with tight monitoring of interictal epileptiform activity.

## 1 Introduction

Following its first application to the human scalp by Berger in the 1920s (Berger, 1931), electroencephalography (EEG) developed rapidly, beginning with discoveries of pathological epileptiform discharges (Gibbs et al., 1935) as well as physiological sleep (Davis et al., 1938) and wake oscillations (Jasper and Carmichael, 1935) that revolutionized our understanding of brain activity. Subsequent milestones in the evolution of EEG (Jasper, 1948) included the digitization of recordings with increased temporal resolution, an increased number of scalp electrodes for better source localization (Brodbeck et al., 2011), and placement of intracranial electrodes (Jasper, 1949) for improved spatial resolution.

Clinical observations led early neurologists to define epilepsy as the recurrence of spontaneous seizures (Gowers, 1881). The advent of EEG bolstered clinical diagnosis of epilepsy, as seizures and interictal discharges could be objectively measured with millisecond precision, but short-duration recordings largely obscured neural dynamics operating over longer timescales. A century later, conventional EEG is widely available but remains highly limited, severely under-sampling long timescale phenomena whose relevance to clinical epilepsy has recently become clear.

**Fig. 1:**
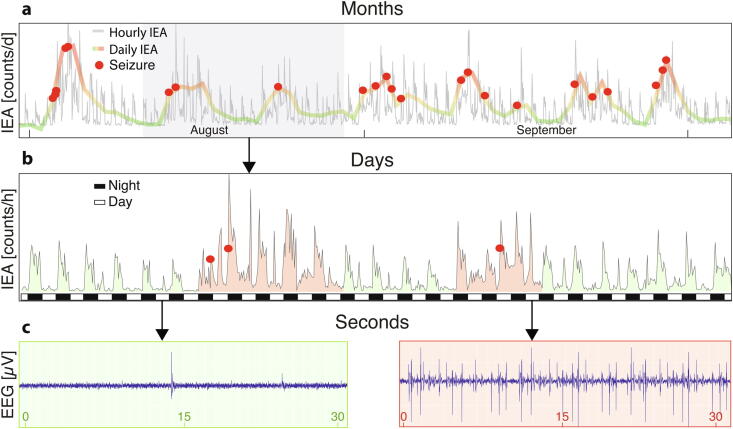
**Fluctuations of epileptic brain activity. a:** months- long recording of chronic EEG in one illustrative subject demonstrating circadian and multidien fluctuations in hourly and daily counts of interictal epileptiform activity (IEA), respectively. **b:** days-long recording in the same subject emphasizing circadian cycles: IEA waxes during nighttime, black rectangles, and wanes during daytime, white rectangles. Starting day 7, an underlying slow oscillation (multidien cycle) increases overall IEA for 5 days before returning to baseline, delineating a pro-ictal state during which two seizures occur. Five days later, another shorter pro-ictal state takes place leading to one seizure. Seizures occur during these periods, indicating heightened seizure risk during these phases of the multidien cycle. **c:** one-minute long EEGs taken at the same hour on different days, reveal strikingly different counts of interictal epileptiform discharges.

We (Baud et al., 2018) and others (Karoly et al., 2016, Maturana et al., 2020, Spencer et al., 2016) have used months- to years-long intracranial EEG recordings to unravel the temporal organization of interictal epileptiform discharges and seizures (Fig. 1). These findings have potentially profound consequences for the practice of clinical electrophysiology in epilepsy. The goal of this review is to challenge current EEG practice in light of recent discoveries on cycles of epileptic brain activity that cannot be observed through short-term recordings. Because seizure timing depends critically on these cycles, we anticipate that EEG practice will evolve towards continuous monitoring of epileptic brain activity, analogous to the recent development of implantable loop recorders for rare cardiac events. Accessing the quantitative era of epilepsy care will require collaboration with the device industry to develop minimally or non-invasive devices that are capable of providing chronic recordings of cycles of epileptic activity.

## 2 Current practice

Classically, epilepsy is a clinical diagnosis and EEG provides supportive information. For example, EEG can help confirm an epileptic etiology for clinical spells and may be useful to monitor for seizure recurrence following medical (Lamberink et al., 2017) or surgical therapy (Rathore and Radhakrishnan, 2010). In current practice, the main uses of EEG can be divided into diagnostic, localizing, and monitoring applications.

### 2.1 Diagnosing epilepsy with EEG

EEG is the most important paraclinical modality to inform a diagnosis of epilepsy. Indeed, recording a seizure on EEG is the most direct proof for epilepsy and enables a refined electroclinical characterization (Beniczky et al., 2016), which often has diagnostic and therapeutic consequences. For example, it can rule out a non-epileptic cause for seizures. EEG often captures interictal epileptiform activity (IEA) in the form of spikes, sharp-waves or other epileptiform discharges that typically do not provoke any symptoms. Thus, in the appropriate clinical context, detection of epileptiform discharges on EEG helps secure the diagnosis of epilepsy (Wirrell, 2010), even in the absence of a recorded seizure.

Routine outpatient EEG typically involves a 20-min recording, which can be repeated every few months, for initial diagnosis of epilepsy or to monitor epileptic brain activity. Diagnostic yield can be increased with “activating methods,” including photic stimulation, hyperventilation, which facilitate the recording of epileptiform activity (Pillai and Sperling, 2006, Schwarz and Zangemeister, 1978). Routine EEG is also often performed after a night of sleep deprivation to favor the emergence of sleep and interictal activity during a 45- to 60-minute recording (Rossi et al., 2020). The practice of routine EEG is cumbersome and requires highly-specialized personnel for electrode application, artifact mitigation, and data recording and handling (Sinha et al., 2016). For 20 min of EEG, no less than 45–60 min are spent in total, from application of electrodes to the scalp until unmounting. The interpretation of a routine EEG by an expert provider takes 5–15 min, depending on the complexity of the traces. Routine EEGs are performed several times per day in University hospitals or in private practice and the technique has been standardized (Jasper, 1958, Klem et al., 1999) and perfected over decades. Guidelines also include recommendations for high-yield montages, such as those including lower temporal electrodes (Seeck et al., 2017).

Inpatient EEG, often practiced on the neurology ward or in specialized epilepsy monitoring units (EMUs), powerfully complements outpatient EEG by enabling longer recordings, including during sleep, and increase the chance of capturing seizures. This practice is utilized either to confirm a diagnosis of epilepsy or for presurgical seizure localization. In recent years, home-based long-term video-EEG solutions have been proposed (Goodwin et al., 2014, Kandler et al., 2017) to mitigate the high costs of inpatient investigations. These solutions help capture rare seizures in a natural environment but cannot go beyond a few days and still require involvement of technicians to maintain EEG signal quality.

### 2.2 Localizing seizures with EEG

When patients with focal epilepsy become pharmacoresistant, an attempt at localizing seizures (Foldvary et al., 2001) and determining the feasibility of resective surgery is indicated. Typically, inpatient EEG is augmented by concurrent video-recordings and dynamic neurological examination in the ictal and/or post-ictal phase for refined electro-clinical correlations (Beniczky et al., 2016, Hamandi et al., 2017). The quality of recordings in this setting hinges upon the continued presence of EEG technicians, who intervene periodically to ensure electrode integrity and minimize artifact (Sinha et al., 2016). Ictal scalp EEG has lateralizing or localizing value, often at the lobar level and sometimes at the sub-lobar level. Other advances have been made to localize the source of epileptiform discharges from scalp signals (Brodbeck et al., 2011) combined with individualized head models (van Mierlo et al., 2017). But when scalp EEG inadequately localizes seizures, intracranial EEG (icEEG) is often unavoidable to record activity directly from brain parenchyma, either with subdural sheets of electrodes placed on the cortical surface or with penetrating depth electrodes (Zijlmans et al., 2019).

### 2.3 Monitoring epileptic brain activity with EEG

#### 2.3.1 Monitoring Interictal epileptiform activity

**Fig. 2:**
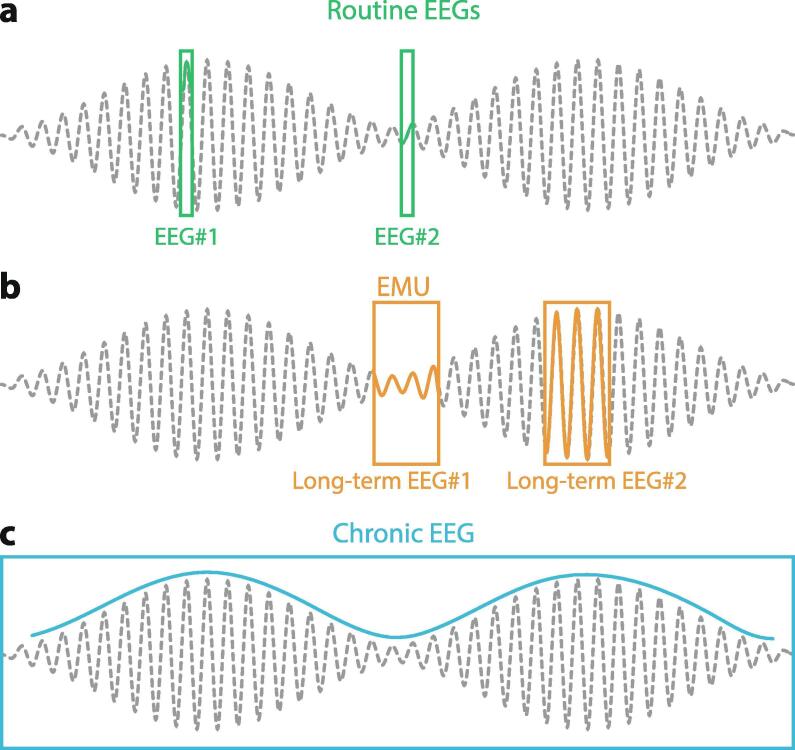
**Interictal epileptiform activity through different temporal lenses. a**: Serial routine EEGs randomly timed at different phases of an underlying cycle reveal very different numbers of interictal epileptiform discharges over the same duration of recording. **b**: Longer term EEGs randomly timed at different phases of an underlying long cycle only capture the shorter cycle (circadian), albeit oscillating with different magnitude. EMU: epilepsy monitoring unit. **c**: Only chronic EEG tracks the short (circadian) and long (multidien) cycles by measuring IEA at all time-points. Grey dotted trace: arbitrary counts of IEA over time undergoing cyclical fluctuations at two timescales.

It has been long suggested that quantifying IEA—for example, counts of discharges per hour, or the duration or frequency of single discharges—could help measure fluctuations in excitability of epileptic cortex (Jasper, 1949). Indeed, IEA fluctuates over time, as can be seen in continuous inpatient EEG at different times of the day (Fig. 2), depending on the momentary vigilance stage, or over the course of the hospital stay, sometimes in relation to pharmacological adjustments. From one routine EEG to the next, the number of recorded discharges may also vary greatly. Active research is ongoing to determine the prognostic value of routine EEGs in regard to seizure recurrence. In the idiopathic generalized epilepsies, three recent studies converged in showing that prolonged epileptiform discharges (Arntsen et al., 2017, Jensen et al., 2019) and the presence of generalized polyspikes in sleep (Jensen et al., 2019, Sun et al., 2018) are markers of drug resistance. Specifically in Juvenile Myoclonic Epilepsy, performing routine EEG in the morning may be more useful than at other times of the day to capture IEA (Labate et al., 2007). In practice, fluctuations in IEA are often used to make momentous clinical decisions, such as gauging response to a new anti-seizure medication (ASM), determining whether a patient is safe to drive, or estimating risk of seizure recurrence after tapering off ASMs. Unfortunately, interpretation of EEG in these settings is severely limited by the intrinsic variability of IEA, and overinterpreting the results of a brief EEG is a common pitfall for clinicians (Fig. 2). Truly monitoring IEA involves a continuous and exhaustive count of interictal epileptiform discharges, as their occurrence is under the combined influences of cycles at multiple timescales (Baud et al., 2018). While scalp EEG is a specific method, it is highly insensitive. Some patients with poorly controlled epilepsy never have epileptiform discharges on scalp EEG, even with serial recordings. The sensitivity of intracranial EEG for discharges is higher (Tao et al., 2005), but some patients show a paucity of discharges while others have near-continuous discharges. Whereas the absolute count of interictal spikes may be a poor marker for seizure burden, their fluctuation over time bears important information, as discussed in Section 3.

#### 2.3.2 Monitoring seizures

For the neurophysiologist, seizures are stereotyped electrographic patterns that unfold in time and space. For patients, seizures are the paroxysmal symptoms that recur sporadically over time. Rarely do these two views match perfectly, and their incongruence is captured by the term, “subclinical seizures,” referring to the fact that the patient did not notice or did not remember the seizure and/or that it was not externally visible (Elger and Hoppe, 2018). However, it is highly questionable whether “subclinical seizures” exist in the true sense (Gotman, 2011), and it might be more prudent to use the term, “electrographic seizure.”

The issue of under-reporting (and over-reporting) seizures has been discussed in the recent literature (Elger and Hoppe, 2018, Karoly et al., 2018). In the opinion of many experts, EEG remains the best starting point to count seizures because it is objective. Symptom burden for patients may be equally important in the context of therapeutic management. While electrographic and self-reported seizures may not match perfectly at the timescale of hours, they bear the same relationship with cycles of IEA, as discussed next. Self-reported seizures may only represent the ‘tip of the iceberg,’ and there is a need to quantify seizures reliably in a natural environment (Quigg et al., 2020).

## 3 Cycles in epilepsy

Historical observations (Bercel, 1964, Gowers, 1881, Griffiths and Fox, 1938, Langdon-Down and Brain, 1929 have established that robust cycles of seizures exist in many people with epilepsy, beyond catamenial seizures in women (Herzog et al., 2015). Recent studies (Baud et al., 2018, Karoly et al., 2016, Maturana et al., 2020, Spencer et al., 2016, Leguia et al., 2021) have unraveled the cyclical nature of IEA and seizures.

### 3.1 Circadian cycle

Circadian modulations in epilepsy have been widely reported in the literature dating back to Gowers in the 19th century and his ‘dirunal’ and ‘nocturnal’ epilepsies (Gowers, 1881). Although not necessarily present on every cycle, seizures have preferential times of occurrence in any given patient. A combined influence of brain states and circadian time is likely, and certain epilepsy syndromes are characterized by seizures arising from sleep (Licchetta et al., 2017). For example, in children with continuous spike-wave during slow-wave sleep, epileptic brain activity is essentially dependent on brain state. In contrast, some people with epilepsy have seizures that consistently occur in the evening but never in the morning, despite the fact that they are awake at both times, pointing to an influence of time of the day or phase of the circadian cycle.

Abrupt changes in IEA are visible with changes in brain states (e.g. falling asleep), and, since the latter are cyclical (sleep-wake cycle), IEA is not uniformly distributed over 24 h. IEA too seems to be under the combined influence of the sleep-wake cycle and the circadian cycle. The timing of ASMs could also play a role but cannot fully explain the phenomenon.

At the circadian level, peak seizure times and peak IEA are not necessarily synchronous and their exact relationship varies on an individual basis (Baud et al., 2018, Karoly et al., 2016, Leguia et al., 2021). Although some authors have postulated an effect of seizure localization on seizure timing (Spencer et al., 2016), there is currently no clear explanation as to why seizures occur at different times in different patients despite the relative similarity in circadian IEA cycles across patients.

### 3.2 Multidien cycle

Using years-long chronic EEG recordings, we found subject-specific multidien cycles of IEA that were robust over time, frequently with periodicity of 7, 15, or 20–30 days (Baud et al., 2018, Leguia et al., 2021). The device used for this study (RNS® System, NeuroPace, Inc.) is a cranially-implanted neurostimulator approved in the U.S. for treatment of certain forms of drug-resistant focal epilepsy (Bergey et al., 2015). Intracranial lead wires enable the device to detect seizures at their source(s) and to deliver responsive electrical stimulation that promotes normalization of brain activity (Sun and Morrell, 2014). This approach has shown promising therapeutic results (Nair et al., 2020), but chronic recordings of brain activity stored by the device also have powerful diagnostic potential. Extending our analyses to more than 200 patients with chronic EEG data collected during the clinical trials of the device, we found that multidien IEA cycles exist in ∼ 60% of patients (Leguia et al., 2021), less prevalent than ubiquitous circadian cycles but just as robust as circadian cycles and occurring equally in men and women. Thus, multidien cycles of IEA cannot be explained only by catamenial effects (Herzog, 2015).

Most importantly, seizures tend to occur when IEA is rising over days (Fig. 1, Fig. 2; Baud et al., 2018, Leguia et al., 2021, Maturana et al., 2020). Thus, these cycles of IEA reflect cycles of varying seizure risk (i.e. the likelihood of a seizure). Seizure risk is heightened during the days when IEA increases, a state termed ‘pro-ictal’ (Baud et al., 2020). Pro-ictal states last ∼3–7 days and can increase relative risk of seizures by 10-fold (Proix et al., 2020). Within these pro-ictal states, peak circadian times for seizures are found, illustrating the shared influence of both cycles on seizure risk. Pro-ictal states likely reflect slow changes in cortical excitability, although direct evidence for this is lacking.

### 3.3 Circannual cycle

A subgroup of subjects also demonstrates increased seizure rates during certain seasons, although the strength of this particular cycle is lower than circadian and multidien seizure cycles (Leguia et al., 2021).

### 3.4 The relationship between IEA and seizures

Earlier studies employing short-term EEG recordings reported apparently conflicting evidence of decreased or increased IEA before seizures (Avoli et al., 2006, Gotman and Marciani, 1985, Janszky et al., 2001, Krishnan et al., 2014), and, similarly, changes in IEA can vary after seizures (Gotman and Marciani, 1985, Janszky et al., 2001). When viewed through a wider temporal lens, the source of this discrepancy becomes evident. Circadian timing of seizures and peak IEA can be different in a patient-specific manner (Baud et al., 2018, Karoly et al., 2016). Thus, a general rule for the behavior of IEA in the hours before and after seizures does not exist at the population level. In sharp contrast, daily IEA that cumulates all events over 24-hours (thereby leveling out circadian variation) systematically increases in the days around seizures across individuals (Baud et al., 2018, Fig. 3). This relationship between rising daily IEA and seizures holds true within and across human subjects from separate cohorts (Baud et al., 2018, Maturana et al., 2020), as well as in animal models of epilepsy (Baud et al., 2019, Gregg et al., 2020).

**Fig. 3:**
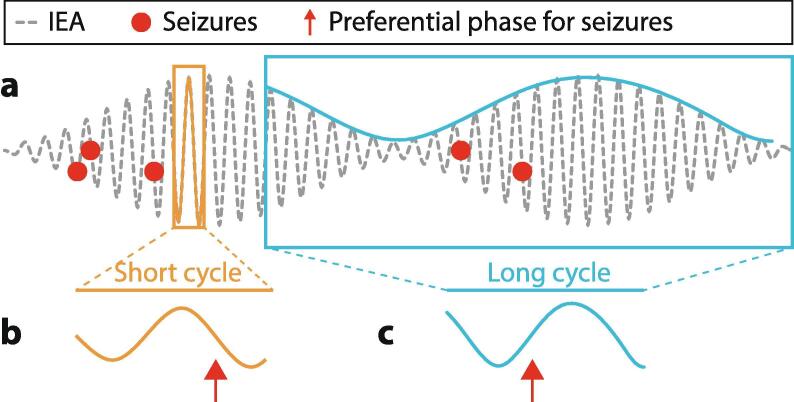
**Cyclical relationship between IEA and seizures. a**: Cyclical fluctuations of IEA (grey dotted line) with intermingled short (orange) and a long (blue) periods. Five seizures (red dots) occur at different times, which corresponds to different phases of the two co-existing cycles. **b**: Expanded average short cycle and preferred seizure timing in the falling phase (arrow). **c**: Condensed average long cycle and preferred seizure timing in the rising phase (arrow).

### 3.5 The precise timing of seizures

The discovery of cycles in epilepsy helps define periods of increased seizure risk. Seizures can occur at any time during these periods, or not occur at all. Seizure timing may be increasingly well-defined when taking into account co-existing cyclical factors that can align temporarily, such as time of day and phase of multidien cycles (Proix et al., 2020). In addition, non-cyclical factors, such as patient-specific seizure provoking factors (e.g. stress, sleep deprivation, ASM non-compliance, etc.) may play a key role in seizure timing during high-risk periods but fail to precipitate a seizure during low-risk periods. In a state permissive for seizures, a very small external perturbation can push brain networks into the ictal state (Baud et al., 2020).

### 3.6 Vanishing IEA

**Fig. 4:**
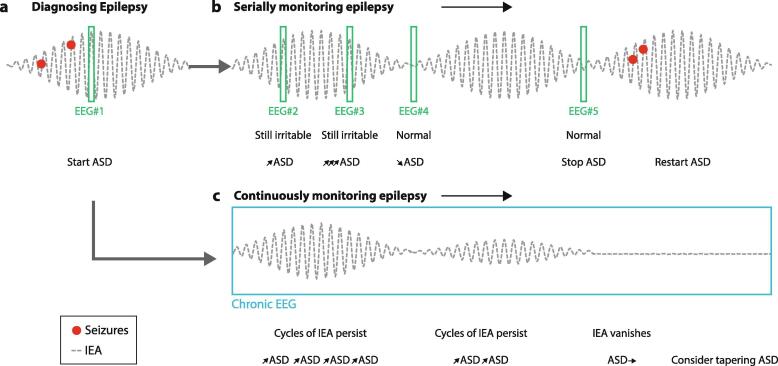
**Monitoring epilepsy with chronic versus serial EEGs. a**: IEA fluctuations (dotted grey line), seizures (red dots), and brief routine EEGs. **b:** Hypothetical scenario, where serial routine EEGs lead to certain interpretations and therapeutic decisions (lower rows) based on too short information. **c**: Hypothetical scenario, where chronic EEG may enable different interpretation leading to smoother therapeutic decisions and better outcome. ASD: anti-seizure drug. The single versus multiple arrows represent smooth vs. abrupt changes, respectively. (For interpretation of the references to colour in this figure legend, the reader is referred to the web version of this article.)

A recent study showed that the addition of ASMs leading to self-reported improvements in seizure burden was paralleled by a decreasing trend in IEA, although rhythmicity persisted (Quraishi et al., 2019). In a more extreme scenario, when ASMs achieve complete seizure freedom, multidien fluctuations of IEA vanish (Fig. 4), suggesting that sustained absence of multidien IEA cycles may be a biomarker of remission in epilepsy (Baud *et al*., in preparation).

## 4 Practical consequences for current practice

Important limitations of recent studies on cycles in epilepsy relate to the fact that they only included patients with drug-resistant focal epilepsy who were implanted with devices for icEEG (Baud et al., 2018, Karoly et al., 2016). Thus, it is not formally established that people with primary generalized epilepsies would have the same trends over several days, but we suspect that this may be the case, as earlier clinical observations on seizure cycles did not distinguish generalized versus focal epilepsies (Gowers, 1881, Griffiths and Fox, 1938, Langdon-Down and Brain, 1929). Also, it is currently unknown whether fluctuations in IEA can be detected extracranially, as scalp EEG typically only reveals a fraction of the epileptiform discharges seen by icEEG (Baumgartner et al., 1995, Tao et al., 2005).

Still, there is clearly a striking mismatch between the temporal windows afforded by most forms of EEG and the timescales over which epilepsy dynamics operate. By analogy, understanding a musical piece by hearing only three notes is difficult; one can hazard a guess at the genre and artist, or even occasionally identify the piece, but understanding the full structure of the verse, chorus, and instrumental accompaniment cannot be achieved with so few elements. This under-sampling has significant clinical implications.

### 4.1 Consequences for the interpretation of routine EEGs

The value of EEG for capturing IEA and supporting a diagnosis of epilepsy is not questioned (Wirrell, 2010). However, the interpretation of changes in IEA in serial 20-minute routine EEGs must be carefully evaluated. For example, observing three versus one interictal epileptiform discharge(s) in serial EEGs and concluding that this reflects clinical worsening is not warranted (Fig. 2). Small (and larger) changes in IEA result from “natural” fluctuations, including the influence of time of the day when the recording was done (Karoly et al., 2016). Scheduling sequential EEGs at the same time of day may even out some fluctuations (Labate et al., 2007) but would not account for those linked to multidien cycles of IEA (Baud et al., 2018). Furthermore, the occurrence of individual epileptiform discharges is best understood as a stochastic process with different rates at different times (Baud et al., 2020). Thus, in our opinion, quantitative changes in IEA on serial routine EEGs have limited clinical value.

One possible exception involves the case when IEA is entirely absent on successive routine EEGs (i.e. IEA tends to zero). Because scalp EEG is insensitive, it is impossible to know if an underlying cycle persists with infrequent epileptiform discharges that are systematically missed (Tao et al., 2005). However, a clear change from high to zero IEA on serial routine EEGs could reflect a favorable change in the dynamics of the epileptic brain. Importantly, the absence of IEA on routine EEG was shown to be a positive predictor for continued seizure freedom after ASM withdrawal (Lamberink et al., 2017). In generalized epilepsy, the duration of sustained interictal epileptiform discharges may be of prognostic factor (Arntsen et al., 2017, Jensen et al., 2019). Whether longer-term EEGs (e.g. 24-hours) may refine this risk-stratification has not been investigated, to our knowledge.

### 4.2 Consequences for the interpretation of inpatient EEG

The goal of inpatient EEG is typically to capture seizures. If the risk of seizures fluctuates cyclically in epilepsy, the diagnostic yield of inpatient EEGs may be determined by their timing. Indeed, non-diagnostic inpatient admissions may result from unfortunate timing when seizure risk is low. If seizure risk was known for an individual, this could increase the yield of inpatient monitoring.

Most inpatient admissions are of sufficient duration (∼3–14 days) to capture circadian cycles of IEA and seizures (van Campen et al., 2015) but usually too short to capture full multidien cycles (∼7–60 days) and characterize their periodicity (Fig. 2). Circadian timing of seizures established during inpatient EEG may not always reflect what patients experience at home. Unless the circadian effect is very strong, peak-times for seizures can only be evaluated in the form of a distribution over 24-hours, requiring a minimum number of dozens of observations for a robust result. Moreover, inpatient epilepsy workup may lead to non-stationarities in cortical excitability, as ASMs are frequently changed to hasten the occurrence of seizures or, by contrast, to rapidly control seizures.

Incomplete sampling of multidien cycles during short inpatient admissions may also confound interpretation of fluctuations in IEA and seizures. For example, a drastic decrease in seizures during an inpatient admission may be attributed to ASM changes but actually relate to the falling phase of a multidien cycle. In light of recent discoveries on cycles in epilepsy (Baud et al., 2018), interpretation of changes in IEA over successive days and correlation with therapeutic changes should be made with caution.

### 4.3 Consequences for treatment titration

Changes in IEA should not be used to guide treatment titration, at least not until the broader phenomenon is more fully understood. Basing treatment decisions on an under-sampled biomarker is problematic because over- and under-treatment could result in side-effects and seizure recurrence, respectively. To smoothly reach a steady state in the epileptic brain—a dynamical system—continuous monitoring of adequately-sampled IEA would enable understanding of changes in real-time and titration of ASMs in rational ways (Fig. 4). These concepts are formally captured in pharmacology (Donnet and Samson, 2013), intensive care medicine (Daun et al., 2008), and control engineering in the form of differential equations. In practical terms, having a biomarker obviates the need to wait for seizures to know whether or not the desired anti-seizure effect was attained (Karoly et al., 2018).

### 4.4 Consequences for prognostication

When IEA cycles over days, seizures recur during certain periods. But, can EEG be informative about long-term seizure freedom? Using chronic icEEG, we recently found that IEA tended to stabilize around zero (or very low values) when medication changes lead to complete seizure freedom (Baud *et al*., in preparation, Fig. 4c). Thus, absent IEA in long-term EEG may be an objective sign of seizure freedom. On the contrary, given that persistence of IEA after surgical treatment is associated with relapses (Rathore and Radhakrishnan, 2010), recurrence of IEA after a long period of seizure freedom might motivate adjustment of ASMs. When chronic EEG is unavailable, we suggest that EEG-based treatment decisions and prognostication should be reserved for clear-cut cases of absent vs. present IEA.

### 4.5 Consequences for driving

In some countries, traffic legislation has included the notion of an “EEG compatible with driving” to deliver a driver’s license to a person with epilepsy (Markhus et al., 2020). Although ‘compatibility’ is subject to the clinician's interpretation, sustained epileptiform discharges lasting a few seconds are typically considered incompatible. Given the cycles of IEA described above, various routine EEG performed in the same patient may be interpreted as compatible with driving when recorded at certain times, and incompatible when recorded at other times. One practical concern in the context of accumulating more data for better epilepsy care is that unraveling the full extent of IEA and, in some cases, electrographic seizures in many people with epilepsy will lead them to lose their driver’s license, a stigmatization that will likely only be avoided with a better understanding of transient cognitive impairments related to ‘subclinical’ epileptiform activity (Kleen and Kirsch, 2017).

## 5 A paradigm shift

Glycemic fluctuation in diabetes can culminate in attacks of altered consciousness, similar to epilepsy. Monitoring of a simple biomarker with high sampling rate—blood glucose, in this case—ensures that values remain under control, as diabetic patients can adjust their medication on that basis.

Tight disease control based on a wealth of objective data is precisely what is lacking in the current approach to epilepsy. Epileptiform discharges are readily identified in EEG, and monitoring them can provide a biomarker for anticipating seizures (Baud et al., 2018, Maturana et al., 2020, Proix et al., 2020) and prognosticating the effects of a new medication (Quraishi et al., 2019. Yet, accurate methods to count seizures are crucially missing from daily neurological practice (Elger and Hoppe, 2018). These limitations reveal an unmet need for constant access to epileptic brain activity so as to leverage long-established biomarkers of epilepsy for clinical care. Currently available scalp EEGs are impractical for this goal, but the design of next-generation recording devices holds considerable promise.

### 5.1 Chronic continuous EEG monitoring

Several centers and companies around the world have developed new systems for chronic EEG. The FDA-approved RNS® System involves a neurostimulator that provides a limited form of electrocorticography constrained by on-board storage limitations. The Percept^TM^ PC device (Medtronic, USA) received FDA approval and CE-labeling in 2020 for thalamic neurostimulation and BrainSense^TM^ technology that provides minutes-long recordings (from thalamus) but lacks a built-in detector for epileptiform discharges. Contrasting with these devices designed primarily for therapeutic applications, other less-invasive technologies are focused on diagnostic applications. The 24/7 EEG^TM^ SubQ recorder (UNEEG^TM^ Medical, Denmark) features two bipolar electrodes and has received CE-labeling in 2019 as the first sub-scalp EEG system for long-term ambulatory monitoring. Four other sub-scalp recording devices are currently in development in the USA, Switzerland, Finland, and Australia (Duun‐Henriksen et al., 2020).

### 5.2 EEG anywhere, anytime

Clinical EEG remains largely a qualitative tool, requiring visual interpretation by an expert. Yet, digital EEG is a method well-suited for quantitative and automated analyses (Baud et al., 2017, Burrello et al., 2018, Gotman et al., 1979, van Mierlo et al., 2017). In addition to recording devices, cloud-based systems that are capable of housing and analyzing vast amounts of data will be needed, and their outputs must be validated by expert annotations of the EEG (Baldassano et al., 2019). This will require a collaborative effort between industry and academia. Future developments will be driven by the need for monitoring the dynamics of epilepsy to improve treatment in a closed-loop paradigm, as successfully implemented for other chronic diseases. People with epilepsy will be empowered to take on active roles, providing feedback on their symptoms and benefiting from direct access to information about their health (Baud and Rao, 2018).

## 6 Conclusion

Epilepsy is a neurological disorder characterized by its temporal dynamics, and growing awareness that epilepsy is a cyclical disorder has consequences for the practice of clinical neurophysiology. Interictal discharges are key biomarkers of epilepsy, however their variable occurrence over time should not be over-interpreted. Electrographic seizures must become the objective gold-standard to count seizures, with emphasis on the symptoms they produce (or the lack thereof) to guide clinical decisions. Smooth titration of ASMs, prognostication of seizure relapses, and forecasts of seizure timing will increasingly become feasible as sufficient data becomes available.

## References

[b0005] Arntsen V., Sand T., Syvertsen M.R., Brodtkorb E. (2017). Prolonged epileptiform EEG runs are associated with persistent seizures in juvenile myoclonic epilepsy. Epilepsy Res..

[b0010] Avoli M., Biagini G., de Curtis M. (2006). Do Interictal Spikes Sustain Seizures and Epileptogenesis?. Epilepsy Curr..

[b0015] Baldassano S., Zhao X., Brinkmann B., Kremen V., Bernabei J., Cook M., Denison T., Worrell G., Litt B. (2019). Cloud computing for seizure detection in implanted neural devices. J. Neural Eng..

[b0020] Baud M.O., Ghestem A., Benoliel J.J., Becker C., Bernard C. (2019). Endogenous multidien rhythm of epilepsy in rats. Exp. Neurol..

[b0025] Baud M.O., Kleen J.K., Anumanchipalli G.K., Hamilton L.S., Tan Y.L., Knowlton R., Chang E.F. (2017). Unsupervised learning of spatiotemporal interictal discharges in focal epilepsy. Neurosurgery.

[b0030] Baud M.O., Kleen J.K., Mirro E.A., Andrechak J.C., King-Stephens D., Chang E.F., Rao V.R. (2018). Multi-day rhythms modulate seizure risk in epilepsy. Nat. Commun..

[b0040] Baud M.O., Proix T., Rao V.R., Schindler K. (2020). Chance and risk in epilepsy. Curr. Opin. Neurol..

[b0045] Baud M.O., Rao V.R. (2018). Gauging seizure risk. Neurology.

[b0050] Baumgartner C., Lindinger G., Ebner A., Aull S., Serles W., Olbrich A., Lurger S., Czech T., Burgess R., Luders H. (1995). Propagation of interictal epileptic activity in temporal lobe epilepsy. Neurology.

[b0055] Beniczky S., Neufeld M., Diehl B., Dobesberger J., Trinka E., Mameniskiene R., Rheims S., Gil-Nagel A., Craiu D., Pressler R., Krysl D., Lebedinsky A., Tassi L., Rubboli G., Ryvlin P. (2016). Testing patients during seizures: A European consensus procedure developed by a joint taskforce of the ILAE – Commission on European Affairs and the European Epilepsy Monitoring Unit Association. Epilepsia.

[b0060] Bercel N.A. (1964). the Periodic Features of Some Seizure States. Ann. N. Y. Acad. Sci..

[b0065] Berger H. (1931). Über das Elektrenkephalogramm des Menschen. Arch. Psychiatr. Nervenkr..

[b0070] Bergey G.K., Morrell M.J., Mizrahi E.M., Goldman A., King-Stephens D., Nair D., Srinivasan S., Jobst B., Gross R.E., Shields D.C., Barkley G., Salanova V., Olejniczak P., Cole A., Cash S.S., Noe K., Wharen R., Worrell G., Murro A.M., Edwards J., Duchowny M., Spencer D., Smith M., Geller E., Gwinn R., Skidmore C., Eisenschenk S., Berg M., Heck C., Van Ness P., Fountain N., Rutecki P., Massey A., O’Donovan C., Labar D., Duckrow R.B., Hirsch L.J., Courtney T., Sun F.T., Seale C.G. (2015). Long-term treatment with responsive brain stimulation in adults with refractory partial seizures. Neurology.

[b0075] Brodbeck V., Spinelli L., Lascano A.M., Wissmeier M., Vargas M.I., Vulliemoz S., Pollo C., Schaller K., Michel C.M., Seeck M. (2011). Electroencephalographic source imaging: A prospective study of 152 operated epileptic patients. Brain.

[b0080] Burrello, A., Schindler, K., Benini, L., Rahimi, A., 2018. One-shot Learning for iEEG Seizure Detection Using End-to-end Binary Operations: Local Binary Patterns with Hyperdimensional Computing. 2018 IEEE Biomed. Circuits Syst. Conf. BioCAS 2018 - Proc. 1–4. 10.1109/BIOCAS.2018.8584751

[b0085] Daun S., Rubin J., Vodovotz Y., Clermont G. (2008). Equation-based models of dynamic biological systems. J. Crit. Care.

[b0090] Davis H., Davis P.A., Loomis A.L., Harvey E.N., Hobart G. (1938). Human brain potentials during the onset of sleep. J. Neurophysiol..

[b0095] Donnet S., Samson A. (2013). A review on estimation of stochastic differential equations for pharmacokinetic/pharmacodynamic models. Adv. Drug Deliv. Rev..

[b0100] Duun-Henriksen J., Baud M., Richardson M.P., Cook M., Kouvas G., Heasman J.M., Friedman D., Peltola J., Zibrandtsen I.C., Kjaer T.W. (2020). A new era in electroencephalographic monitoring? Subscalp devices for ultra–long-term recordings. Epilepsia.

[b0105] Elger C.E., Hoppe C. (2018). Diagnostic challenges in epilepsy: seizure under-reporting and seizure detection. Lancet Neurol..

[b0110] Foldvary N., Klem G., Hammel J., Bingaman W., Najm I., Lüders H. (2001). The localizing value of ictal EEG in focal epilepsy. Neurology.

[b0115] Gibbs F.A., Davis H., Lennox W.G. (1935). The electro-encephalogram in epilepsy and in conditions of impaired consciousness. Arch. Neurol. Psychiatry.

[b0120] Goodwin E., Kandler R.H., Alix J.J.P. (2014). The value of home video with ambulatory EEG: A prospective service review. Seizure.

[b0125] Gotman J. (2011). A few thoughts on “What is a seizure?”. Epilepsy Behav..

[b0130] Gotman J., Ives J.R., Gloor P. (1979). Automatic recognition of inter-ictal epileptic activity in prolonged EEG recordings. Electroencephalogr. Clin. Neurophysiol..

[b0135] Gotman J., Marciani M.G. (1985). Electroencephalographic spiking activity, drug levels, and seizure occurence in epileptic patients. Ann. Neurol..

[b0140] Gowers W.R. (1881). Epilepsy and Other Chronic Convulsive Diseases; Their Causes.

[b0145] Gregg N.M., Nasseri M., Kremen V., Patterson E.E., Sturges B.K., Denison T.J., Brinkmann B.H., Worrell G.A. (2020). Circadian and multiday seizure periodicities, and seizure clusters in canine epilepsy. Brain Commun..

[b0150] Griffiths G., Fox J.T. (1938). Rhythm in Epilepsy. Lancet.

[b0155] Hamandi K., Beniczky S., Diehl B., Kandler R.H., Pressler R.M., Sen A., Solomon J., Walker M.C., Bagary M. (2017). Current practice and recommendations in UK epilepsy monitoring units. Report of a national survey and workshop. Seizure.

[b0160] Herzog A.G. (2015). Catamenial epilepsy: Update on prevalence, pathophysiology and treatment from the findings of the NIH Progesterone Treatment Trial. Seizure.

[b0165] Herzog A.G., Fowler K.M., Sperling M.R., Massaro J.M. (2015). Distribution of seizures across the menstrual cycle in women with epilepsy. Epilepsia.

[b0170] Janszky J., Fogarasi A., Jokeit H., Schulz R., Hoppe M., Ebner A. (2001). Spatiotemporal relationship between seizure activity and interictal spikes in temporal lobe epilepsy. Epilepsy Res..

[b0175] Jasper H.H. (1958). The Ten-Twenty Electrode System of the International Federation. Electroencephalogr Clin Neurophysiol..

[b0180] Jasper H.H. (1949). Electrical signs of epileptic discharge. Electroencephalogr. Clin. Neurophysiol..

[b0185] Jasper H.H. (1948). Charting the sea of brain waves. Science..

[b0190] Jasper H.H., Carmichael L. (1935). Electrical potentials from the intact human brain. Science..

[b0195] Jensen C.D., Gesche J., Krøigård T., Beier C.P. (2019). Prognostic Value of Generalized Polyspike Trains and Prolonged Epileptiform EEG Runs. J. Clin. Neurophysiol. Publish Ah.

[b0200] Kandler R., Ponnusamy A., Wragg C. (2017). Video ambulatory EEG: A good alternative to inpatient video telemetry?. Seizure.

[b0205] Karoly P., Goldenholz D.M., Cook M. (2018). Are the days of counting seizures numbered?. Curr. Opin. Neurol..

[b0210] Karoly P.J., Freestone D.R., Boston R., Grayden D.B., Himes D., Leyde K., Seneviratne U., Berkovic S., O’Brien T., Cook M.J. (2016). Interictal spikes and epileptic seizures: Their relationship and underlying rhythmicity. Brain.

[b0215] Kleen J.K., Kirsch H.E. (2017). The nociferous influence of interictal discharges on memory. Brain.

[b0220] Klem G.H., Lüders H.O., Jasper H.H., Elger C. (1999). The ten-twenty electrode system of the International Federation. The International Federation of Clinical Neurophysiology. Electroencephalogr. Clin. Neurophysiol. Suppl..

[b0225] Krishnan B., Vlachos I., Faith A., Mullane S., Williams K., Alexopoulos A., Iasemidis L. (2014). A novel spatiotemporal analysis of peri-ictal spiking to probe the relation of spikes and seizures in epilepsy. Ann. Biomed. Eng..

[b0230] Labate A., Ambrosio R., Gambardella A., Sturniolo M., Pucci F., Quattrone A. (2007). Usefulness of a morning routine EEG recording in patients with juvenile myoclonic epilepsy. Epilepsy Res..

[b0235] Lamberink H.J., Otte W.M., Geerts A.T., Pavlovic M., Ramos-Lizana J., Marson A.G., Overweg J., Sauma L., Specchio L.M., Tennison M., Cardoso T.M.O., Shinnar S., Schmidt D., Geleijns K., Braun K.P.J. (2017). Individualised prediction model of seizure recurrence and long-term outcomes after withdrawal of antiepileptic drugs in seizure-free patients: a systematic review and individual participant data meta-analysis. Lancet Neurol..

[b0240] Langdon-Down M., Brain W.R. (1929). Time of Day in Relation to Convulsions in Epilepsy. Lancet.

[b9000] Leguia M.G., Andrzejak R.G., Rummel C., Fan J.M., Mirro E.A., Tcheng T.K., Rao V.R., Baud M.O. (2021). Seizure cycles in focal epilepsy, JAMA. Neurology.

[b0245] Licchetta L., Bisulli F., Vignatelli L., Zenesini C., Di Vito L., Mostacci B., Rinaldi C., Trippi I., Naldi I., Plazzi G., Provini F., Tinuper P. (2017). Sleep-related hypermotor epilepsy. Neurology.

[b0250] Markhus R., Henning O., Molteberg E., Hećimović H., Ujvari A., Hirsch E., Rheims S., Surges R., Malmgren K., Rüegg S., Gil-Nagel A., Roivainen R., Picard F., Steinhoff B., Marusic P., Mostacci B., Kimiskidis V.K., Mindruta I., Jagella C., Mameniškienė R., Schulze-Bonhage A., Rosenow F., Kelemen A., Fabo D., Walker M.C., Seeck M., Kraemer G., Arsene O.T., Krestel H., Lossius M. (2020). EEG in fitness to drive evaluations in people with epilepsy — Considerable variations across Europe. Seizure.

[b0255] Maturana M.I., Meisel C., Dell K., Karoly P.J., D’Souza W., Grayden D.B., Burkitt A.N., Jiruska P., Kudlacek J., Hlinka J., Cook M.J., Kuhlmann L., Freestone D.R. (2020). Critical slowing down as a biomarker for seizure susceptibility. Nat. Commun..

[b0260] Nair D.R., Laxer K.D., Weber P.B., Murro A.M., Park Y.D., Barkley G.L., Smith B.J., Gwinn R.P., Doherty M.J., Noe K.H., Zimmerman R.S., Bergey G.K., Anderson W.S., Heck C., Liu C.Y., Lee R.W., Sadler T., Duckrow R.B., Hirsch L.J., Wharen R.E., Tatum W., Srinivasan S., McKhann G.M., Agostini M.A., Alexopoulos A.V., Jobst B.C., Roberts D.W., Salanova V., Witt T.C., Cash S.S., Cole A.J., Worrell G.A., Lundstrom B.N., Edwards J.C., Halford J.J., Spencer D.C., Ernst L., Skidmore C.T., Sperling M.R., Miller I., Geller E.B., Berg M.J., Fessler A.J., Rutecki P., Goldman A.M., Mizrahi E.M., Gross R.E., Shields D.C., Schwartz T.H., Labar D.R., Fountain N.B., Elias W.J., Olejniczak P.W., Villemarette-Pittman N.R., Eisenschenk S., Roper S.N., Boggs J.G., Courtney T.A., Sun F.T., Seale C.G., Miller K.L., Skarpaas T.L., Morrell M.J. (2020). Nine-year prospective efficacy and safety of brain-responsive neurostimulation for focal epilepsy. Neurology.

[b0265] Pillai J., Sperling M.R. (2006). Interictal EEG and the diagnosis of epilepsy. Epilepsia.

[b9005] Proix T., Truccolo W., Leguia M.G., Tcheng T.K., King-Stephens D., Rao V.R., Baud M.O. (2020). Forecasting seizure risk in adults with focal epilepsy: a development and validation study. The Lancet Neurology.

[b0270] Quigg M., Skarpaas T.L., Spencer D.C., Fountain N.B., Jarosiewicz B., Morrell M.J. (2020). Electrocorticographic events from long-term ambulatory brain recordings can potentially supplement seizure diaries. Epilepsy Res..

[b0275] Quraishi I.H., Mercier M.R., Skarpaas T.L., Hirsch L.J. (2019). Early detection rate changes from a brain-responsive neurostimulation system predict efficacy of newly added antiseizure drugs. Epilepsia.

[b0280] Rathore C., Radhakrishnan K. (2010). Prognostic significance of interictal epileptiform discharges after epilepsy surgery. J. Clin. Neurophysiol..

[b0285] Rossi K.C., Joe J., Makhija M., Goldenholz D.M. (2020). Insufficient Sleep, Electroencephalogram Activation, and Seizure Risk: Re-Evaluating the Evidence. Ann. Neurol..

[b0290] Schwarz J.R., Zangemeister W.H. (1978). The diagnostic value of the short sleep EEG and other provocative methods following sleep deprivation. J. Neurol..

[b0295] Seeck M., Koessler L., Bast T., Leijten F., Michel C., Baumgartner C., He B., Beniczky S. (2017). The standardized EEG electrode array of the IFCN. Clin. Neurophysiol..

[b0300] Sinha S.R., Sullivan L., Sabau D., San-Juan D., Dombrowski K.E., Halford J.J., Hani A.J., Drislane F.W., Stecker M.M. (2016). American Clinical Neurophysiology Society Guideline 1: Minimum Technical Requirements for Performing Clinical Electroencephalography. J. Clin. Neurophysiol..

[b0305] Spencer D.C., Sun F.T., Brown S.N., Jobst B.C., Fountain N.B., Wong V.S.S., Mirro E.A., Quigg M. (2016). Circadian and ultradian patterns of epileptiform discharges differ by seizure-onset location during long-term ambulatory intracranial monitoring. Epilepsia.

[b0310] Sun F.T., Morrell M.J. (2014). The RNS System: Responsive cortical stimulation for the treatment of refractory partial epilepsy. Expert Rev. Med. Devices.

[b0315] Sun Y., Seneviratne U., Perucca P., Chen Z., Tan M.K., O’Brien T.J., D’Souza W., Kwan P. (2018). Generalized polyspike train An EEG biomarker of drug-resistant idiopathic generalized epilepsy. Neurology.

[b0320] Tao J.X., Ray A., Hawes-Ebersole S., Ebersole J.S. (2005). Intracranial EEG substrates of scalp EEG interictal spikes. Epilepsia.

[b0325] van Campen J.S., Valentijn F.A., Jansen F.E., Joëls M., Braun K.P.J. (2015). Seizure occurrence and the circadian rhythm of cortisol: A systematic review. Epilepsy Behav..

[b0330] van Mierlo P., Strobbe G., Keereman V., Birot G., Gadeyne S., Gschwind M., Carrette E., Meurs A., Van Roost D., Vonck K., Seeck M., Vulliémoz S., Boon P. (2017). Automated long-term EEG analysis to localize the epileptogenic zone. Epilepsia Open.

[b0335] Wirrell E.C. (2010). Prognostic significance of interictal epileptiform discharges in newly diagnosed seizure disorders. J. Clin. Neurophysiol..

[b0340] Zijlmans M., Zweiphenning W., van Klink N. (2019). Changing concepts in presurgical assessment for epilepsy surgery. Nat. Rev. Neurol..

